# Supplementary material for: Immunogenicity and safety of heterologous boost immunization with PastoCovac Plus against COVID-19 in ChAdOx1-S or BBIBP-CorV primed individuals
Source: PLoS Pathog. 2023 Nov 1;19(11):e1011744. doi: 10.1371/journal.ppat.1011744 (PMC10619776; doi:10.1371/journal.ppat.1011744)
Supplement: S1 Table — (DOCX) [file ppat.1011744.s002.docx]

| **S1 Table. Association of age and history of COVID-19 infection with mean rise and fourfold rise rate of specific antibodies between the ChAdOx1-S primed groups** | | | | |  |
| --- | --- | --- | --- | --- | --- |
|  | **n** | **Homologous ChAdOx1-S** | **n** | **Heterologous**  **ChAdOx1-S /PastoCovac Plus** |  |
| **Anti-spike IgG Fourfold rise** _n (%)_ |  |  |  |  |  |
| **COVID-19 History** |  |  |  |  |  |
| Yes | 14 | 6 (46.2) | 27 | 11 (40.7) |  |
| No | 13 | 5 (35.7) | 40 | 25 (62.5) |  |
| *P* value |  | 0.581* |  | 0.080* |  |
| **Age** |  |  |  |  |  |
| ≥ 50 Years | 4 | 1 (25.0) | 17 | 8 (47.1) |  |
| < 50 Years | 23 | 10 (43.5) | 50 | 28 (56.0) |  |
| *P* value |  | 0.624** |  | 0.523* |  |
| **Anti-spike IgG Rise** _GMT (95% CI)_ |  |  |  |  |  |
| **COVID-19 History** |  |  |  |  |  |
| Yes | 14 | 72.6 (53.7, 98.2) | 27 | 502.9 (220.1, 785.8) |  |
| No | 13 | 72.3 (42.7, 122.6) | 40 | 175.3 (114.9, 267.4) |  |
| *P* value |  | 0.7341^§^ |  | 0.4741^§^ |  |
| **Age** |  |  |  |  |  |
| ≥ 50 Years | 4 | 72.8 (-) | 17 | 226.5 (93.8, 546.5) |  |
| < 50 Years | 23 | 72.5 (52.8, 99.4) | 50 | 190.2 (140.4, 257.5) |  |
| *P* value |  | 0.8914^§^ |  | 0.9197^§^ |  |
| **Neutralizing Ab** **Fourfold rise** _n (%)_ |  |  |  |  |  |
| **COVID-19 History** |  |  |  |  |  |
| Yes | 14 | 4 (28.6) | 27 | 0 (0) |  |
| No | 13 | 3 (23.1) | 40 | 18 (45.0) |  |
| *P* value |  | 0.546** |  | **<0.0001**** |  |
| **Age** |  |  |  |  |  |
| ≥ 50 Years | 4 | 1 (25.0) | 17 | 1 (5.9) |  |
| < 50 Years | 23 | 6 (26.1) | 50 | 17 (34.0) |  |
| *P* value |  | 0.731** |  | **0.028**** |  |
| **Neutralizing Ab Rise** _GMT (95% CI)_ |  |  |  |  |  |
| **COVID-19 History** |  |  |  |  |  |
| Yes | 14 | 7.6 (3.1, 18.5) | 25 | 3.9 (2.2, 6.9) |  |
| No | 13 | 7.1 (3.1, 16.8) | 39 | 12.3 (7.9, 19.1) |  |
| *P* value |  | 0.7709^§^ |  | **0.0002^§^** |  |
| **Age** |  |  |  |  |  |
| ≥ 50 Years | 4 | 12.4 (-) | 16 | 3.3 (1.4, 7.8) |  |
| < 50 Years | 23 | 6.7 (3.4, 13.2) | 48 | 10.5 (7.2, 15.4) |  |
| *P* value |  | 0.7848^§^ |  | **0.0063^§^** |  |

* Pearson Chi-Square, ** Fisher’s Exact Test, § Mann Whitney U.
Bold p values are indicated statistically significant.
