# Supplementary material for: Immunogenicity and safety of heterologous boost immunization with PastoCovac Plus against COVID-19 in ChAdOx1-S or BBIBP-CorV primed individuals
Source: PLoS Pathog. 2023 Nov 1;19(11):e1011744. doi: 10.1371/journal.ppat.1011744 (PMC10619776; doi:10.1371/journal.ppat.1011744)
Supplement: S2 Table — (DOCX) [file ppat.1011744.s003.docx]

| **S2 Table. Association of age and history of COVID-19 infection with mean rise and seroconversion rate of anti-spike IgG antibodies between the BBIBP-CorV -primed groups.** | | | | |
| --- | --- | --- | --- | --- |
|  | **n** | **Homologous BBIBP-CorV** | **n** | **Heterologous**  **BBIBP CorV**  **/PastoCovac Plus** |
| **Anti-spike IgG Seroconversion** _n (%)_ |  |  |  |  |
| **COVID-19 History** |  |  |  |  |
| Yes | 12 | 2 (16.7) | 21 | 19 (90.5) |
| No | 38 | 11 (29.0) | 29 | 27 (93.1) |
| *P* value |  | 0.480** |  | 0.564** |
| **Age** |  |  |  |  |
| ≥ 50 Years | 18 | 7 (38.9) | 24 | 23 (95.8) |
| < 50 Years | 32 | 6 (18.8) | 26 | 23 (88.5) |
| *P* value |  | 0.119* |  | 0.611** |
| **Anti-spike IgG Rise** _GMT (95% CI)_ |  |  |  |  |
| **COVID-19 History** |  |  |  |  |
| Yes | 12 | 56.9 (14.9, 217.4) | 21 | 162.2 (99.7, 263.9) |
| No | 38 | 27.6 (13.8, 55.1) | 29 | 171.5 (120.4, 244.2) |
| *P* value |  | 0.275^§^ |  | 0.913^§^ |
| **Age** |  |  |  |  |
| ≥ 50 Years | 18 | 26.4 (10.8, 64.4) | 24 | 184.0 (115.4, 293.5) |
| < 50 Years | 32 | 37.1 (16.3, 84.6) | 26 | 153.7 (108.5, 217.7) |
| *P* value |  | 0.693^§^ |  | 0.478^§^ |
| **Neutralizing Ab** **Seroconversion** _n (%)_ |  |  |  |  |
| **COVID-19 History** |  |  |  |  |
| Yes | 12 | 0 (0) | 21 | 9 (42.9) |
| No | 38 | 2 (5.3) | 29 | 19 (65.5) |
| *P* value |  | 0.574** |  | 0.111** |
| **Age** |  |  |  |  |
| ≥ 50 Years | 18 | 2 (11.1) | 24 | 15 (62.5) |
| < 50 Years | 32 | 0 (0) | 26 | 13 (50.0) |
| *P* value |  | 0.125* |  | 0.374* |
| **Neutralizing Ab Rise** _GMT (95% CI)_ |  |  |  |  |
| **COVID-19 History** |  |  |  |  |
| Yes | 12 | 0.2 (0.1, 0.8) | 21 | 11.9 (6.3, 22.3) |
| No | 37 | 2.1 (1.0, 4.3) | 29 | 19.2 (12.9, 28.6) |
| *P* value |  | **0.0096^§^** |  | 0.1252^§^ |
| **Age** |  |  |  |  |
| ≥ 50 Years | 17 | 1.6 (0.4, 6.5) | 24 | 16.4 (10.3, 26.0) |
| < 50 Years | 32 | 1.0 (0.5, 2.2) | 26 | 15.1 (8.8, 25.8) |
| *P* value |  | 0.6204^§^ |  | 0.7486^§^ |

* Pearson Chi-Square ** , Fisher’s Exact Test, § Mann Whitney U.
Bold p value are indicated statistically significant.
