# Supplementary material for: Immunogenicity and safety of heterologous boost immunization with PastoCovac Plus against COVID-19 in ChAdOx1-S or BBIBP-CorV primed individuals
Source: PLoS Pathog. 2023 Nov 1;19(11):e1011744. doi: 10.1371/journal.ppat.1011744 (PMC10619776; doi:10.1371/journal.ppat.1011744)
Supplement: S3 Table — (DOCX) [file ppat.1011744.s004.docx]

| **S3 Table. Adverse events data of following the homologous and heterologous booster vaccination.** | | | | | | |
| --- | --- | --- | --- | --- | --- | --- |
|  | **ChAdOx1-S (AstraZeneca)** | |  | **BBIBP-CorV** (**Sinopharm)** | |  |
|  | **Homologous ChAdOx1-S**  **n (%)** | **Heterologous**  **ChAdOx1-S /PastoCovac Plus**  **n (%)** | ***p-*value** | **Homologous BBIBP-CorV**  **n (%)** | **Heterologous**  **BBIBP CorV**  **/PastoCovac Plus**  **n (%)** | ***p-* value** |
| **Local** |  |  |  |  |  |  |
| Local Pain | 12/27 (44.4) | 20/67 (29.9) | 0.230 | 9/50 (18.0) | 10/50 (20.0) | 0.799 |
| **Systemic** |  |  |  |  |  |  |
| Fatigue/Weakness | 13/27 (48.2) | 6/67 (9.0) | **<0.0001** | 5/50 (10.0) | 7/50 (14.0) | 0.760 |
| Fever | 3/27 (11.1) | 1/67 (1.5) | 0.070 | 1/50 (2.0) | 0/50 (0) | 0.500 |
| Nausea | 0/27 (0) | 0/67 (0) | - | 0/50 (0) | 0/50 (0) | - |
| Myalgia | 7/27 (25.9) | 4/67 (6.0) | **0.012** | 1/50 (2.0) | 3/50 (6.0) | 0.617 |
| Headache | 3/27 (11.1) | 3/67 (4.5) | 0.349 | 6/50 (12.0) | 1/50 (2.0) | 0.112 |
| Chills | 4/27 (14.8) | 0/67 (0) | **0.006** | 0/50 (0) | 0/50 (0) | - |
| Anorexia | 0/27 (0) | 0/67 (0) | - | 0/50 (0) | 0/50 (0) | - |
| Ear Ache | 0/27 (0) | 0/67 (0) | - | 0/50 (0) | 0/50 (0) | - |
| Sore throat | 0/27 (0) | 0/67 (0) | - | 0/50 (0) | 0/50 (0) | - |

Bold p values are indicated statistically significant.
