# Supplementary material for: Immunogenicity and safety of heterologous boost immunization with PastoCovac Plus against COVID-19 in ChAdOx1-S or BBIBP-CorV primed individuals
Source: PLoS Pathog. 2023 Nov 1;19(11):e1011744. doi: 10.1371/journal.ppat.1011744 (PMC10619776; doi:10.1371/journal.ppat.1011744)
Supplement: Attachment — Submitted filename: Reviewers Response.docx [file ppat.1011744.s005.docx]

Dear Editor-in-Chief

We do appreciate the great attention of you and the respected reviewers which undoubtedly led to improved quality of the present research.

The manuscript has been fully revised including the text, figures and the tables according the remarks of the reviewers. The discussion part has been subsequently updated.

All the data are deposited within the manuscript and supplementary files. The data used to generate Figures 2 and 3 are also uploaded to the journal editorial management system.

All the changes in the text are shown in green highlighted text and the answers to the reviewers’ comments are provided in blue.

We believe that the revised version of the manuscript has been improved and hope that the revisions meet your expectations. We are ready to take any needed action if necessary.

Sincerely yours,

Prof. Amitis Ramezani

Corresponding Author

Answers to the reviewers’ comments.

Major comments:

• Please add P-values to the figures to indicate significance (or lack thereof) within and between groups.

- **Authors’ response**: Thank you for the comment. The *P* values have been added to the Figures.

• Some individuals were infected during the course of the study and it is not clear if these were excluded or included in the final analysis.

- **Authors’ response**: We would like to acknowledge that we have presented the results both in the crude format and in subgroups of positive and negative COVID-19 -19 infection history. In regression models, we have also provided both the crude estimates and the estimates adjusted for COVID-19 -19 infection history. Please see Figures 2 and 3, Tables 2, 3, and 4, as well as supplementary Table 2.

• The neutralization assay is unknown to me and the data seem odd. Is there an upper limit to this assay? What kind of assay is it, a pseudovirus neutralization assay? Is it possible to provide the serum dilutions that resulted in 50% or 100% virus neutralization? The data is very tight at the top and I wonder if you are losing resolution.

- **Authors’ response**: Thank you for your comment. The neutralizing Ab in the manuscript is an ELISA assay, SARS-CoV-2 Neutralizing Ab (Pishtazteb, Iran) based on RBD-SARS-CoV-2 coated Ags to detect neutralizing Abs against RBD. The Cut-Off is 2.5 µg/ml and all the samples ≥2.5 was considered positive. At the time of the submission, cVNT test was not done but fortunately, it has been done during the review process according to the institute’s ongoing research. So the results are now included in Fig.4.

Moreover, Fig.3 was in logarithmic scale. We provided a better resolution in this Figure by transforming the scale of the figure to linear.

• The recombinant protein vaccine can be described a bit more in the manuscript. It is composed of the RBD alone, which can affect the data in Figure 2 where you are measuring the antibody response to the entire Spike protein.

- **Authors’ response**: Thank you for the great attention. The vaccine has just been fully described in the Introduction section in terms of the primary and booster doses.

• Line 264 you mention a that "PastoCovac Plus leads to a significant anti-spike IgG mean rise compare to the homologous booster recipients". What is this statement based on? Based on Figure 2, I find that hard to be true for the ChAdOx1-S groups.

- **Authors’ response**: The results have been modified according to the new analysis. However, According to Table 2, Anti-IgG mean rise before the booster shot in homologous ChAdOx1-S was 36.4 (26.0, 50.9) and increased to 123.5 (98.7, 154.4). For the heterologous ChAdOx1-S /PastoCovac Plus®, Anti-IgG mean rise before the booster was 64.1 (46.9, 87.5) and increased to 310.5 (232.9, 413.8) and the difference between the two booster types has been estimated significant (*P* <0.0001).

Reviewer #1: The different vaccine types that study participants have received need to be better described. Along the same line, the results should be discussed in light of the different vaccine platforms that were used (adenovirus, recombinant protein, inactivated virus vaccine + adjuvant).

- **Authors’ response**: Thank you for your comment. The vaccine groups have been described more in the method section and the result are now based on the type of the booster shots.

  The potential impact of COVID-19 history is not clearly represented. Which samples in Figures 2 and 3 are from individuals with previous COVID-19 exposure. A color coding system might help here.
- **Authors’ response**: Thank you for your point. The original Figures presented all participants regardless of their COVID-19 infection history. To provide a better understanding of the effect of COVID-19, we have added new panels to the Figures 2 and 3, showing the antibody titers for individuals with and without COVID-19 infection history. The color-coding system is also added to the Figures.

Why are the antibody titers (both ELISA and neutralization) before boost so different between the homologous and heterologous boosted groups for the BBIBP-CoV groups? The differences between these pre-booster titers are even significant according to Table 2. The fold rise in titers in boost is the heterologous boost group is large but the initial pre-boost titers are very low as well.

- **Authors’ response**: Thank you for your point. As the research was conducted through an observational cohort study design, it was anticipated that there would be some variations in the initial titers. Such variations are difficult to control for during the study-design phase. However, in our regression models, we accounted for these pre-booster titer variations and modified the findings accordingly. Please see Tables 2, 3, and 4.

Sup. Table 1: COVID-19 History for the homologous ChadOx1-S: n=13 for Yes and 14 for No. These numbers are the opposite in the other parts of the column for the same samples. Are these numbers swapped by accident?

- **Authors’ response**: Thank you. Yes, the numbers swapped by accident. Correct numbers are as follow, which are also obvious in other rows of the table:

COVID-19 History: Yes: 14

COVID-19 History: No: 13

We corrected it in the revised version of the manuscript.

Since there seems to be COVID-19 history available during and after the study time points, the authors should elaborate more on vaccine efficacy/correlation with observed antibody titers and protection from infection.

- **Authors’ response**: Thank you for your comment. We have presented all our main results both in the crude format and in subgroups of positive and negative Covid-19 infection history. In regression models that compared different booster types, we have also provided both the crude estimates and the estimates adjusted for COVID-19 infection history. Please see Figures 2 and 3, Tables 2, 3, and 4, as well as supplementary Table 2. We also elaborated on this in the Results section (lines 265-271). Further exploration of the effect of COVID-19 infection on antibody titers or the protective impact of boosters against infection was not the study's primary goal, and was not feasible due to the study's limited duration and the low statistical power resulting from a small number of recent COVID-19 infections. In our earlier research, we have addressed this question using a long follow-up period (please see references 1 and 2 below)

Reviewer #2: An ELISA against the RBD alone could be useful and re-analyzing or re-doing the virus neutralization assay

- **Authors’ response**: This is exactly right. Fortunately, the cVNT test has been done during the review process and according to the institute ongoing research and the results are now included.

1. Mostafavi  E, Eybpoosh S, Karamouzian M, Khalili M, Haji Maghsoudi S, Salehi-Vaziri M, et al. Efficacy and safety of a protein-based SARS-Cov-2 vaccine (SOBERANA 02 and SOBERANA Plus): a double-blind, randomized placebo-controlled, phase 3 trial in Iran. **Trial Registration**  isrctn.org Identifier: [IRCT20210303050558N1](https://www.irct.ir/trial/54833)
2. Mona Sadat Larijani, Rahim Sorouri, Sana Eybpoosh, et al. Assessment of long-term adverse events regarding different COVID-19 vaccine regimens within an 18-month follow-up study.
